# Diversity and relative abundance of the bacterial pathogen, *Flavobacterium spp.*, infecting reproductive ecotypes of kokanee salmon

**DOI:** 10.1186/1756-0500-7-778

**Published:** 2014-11-04

**Authors:** Matthew A Lemay, Michael A Russello

**Affiliations:** University of British Columbia, Okanagan Campus, 3247 University Way, Kelowna, BC V1V 1V7 Canada

**Keywords:** Bacteria, Bacterial coldwater disease, *Flavobacterium*, Pathogens, Kokanee, Sockeye salmon, *Oncorhynchus nerka*

## Abstract

**Background:**

Understanding the distribution and abundance of pathogens can provide insight into the evolution and ecology of their host species. Previous research in kokanee, the freshwater form of sockeye salmon (*Oncorhynchus nerka*), found evidence that populations spawning in streams may experience a greater pathogen load compared with populations that spawn on beaches. In this study we tested for differences in the abundance and diversity of the gram-negative bacteria, *Flavobacterium spp*., infecting tissues of kokanee in both of these spawning habitats (streams and beaches). Molecular assays were carried out using primers designed to amplify a ~200 nucleotide region of the gene encoding the ATP synthase alpha subunit (*AtpA*) within the genus *Flavobacterium.* Using a combination of DNA sequencing and quantitative PCR (qPCR) we compared the diversity and relative abundance of *Flavobacterium AtpA* amplicons present in DNA extracted from tissue samples of kokanee collected from each spawning habitat.

**Results:**

We identified 10 *Flavobacterium AtpA* haplotypes among the tissues of stream-spawning kokanee and seven haplotypes among the tissues of beach-spawning kokanee, with only two haplotypes shared between spawning habitats. Haplotypes occurring in the same clade as *F. psychrophilum* were the most prevalent (92% of all reads, 60% of all haplotypes), and occurred in kokanee from both spawning habitats (streams and beaches). Subsequent qPCR assays did not find any significant difference in the relative abundance of *Flavobacterium AtpA* amplicons between samples from the different spawning habitats.

**Conclusions:**

We confirmed the presence of *Flavobacterium spp.* in both spawning habitats and found weak evidence for increased *Flavobacterium* diversity in kokanee sampled from stream-spawning sites. However, the quantity of *Flavobacterium* DNA did not differ between spawning habitats. We recommend further study aimed at quantifying pathogen diversity and abundance in population-level samples of kokanee combined with environmental sampling to better understand the ecology of pathogen infection in this species.

**Electronic supplementary material:**

The online version of this article (doi:10.1186/1756-0500-7-778) contains supplementary material, which is available to authorized users.

## Background

Pathogens can play an important role in the evolution of their hosts [1–3]. This can occur when variation in pathogen diversity over small spatial or temporal scales imposes divergent selection on populations of their host species [1, 4]. In salmonids, for example, genetic diversity associated with the major histocompatability complex can vary at micro-geographical scales [1, 5], reflecting local adaptation in response to heterogeneous pathogen regimes [2].

Kokanee, the freshwater form of sockeye salmon (*Oncorhynchus nerka*), occurs as two reproductive ecotypes, which differ in their choice of spawning habitat (streams vs. beaches) [6, 7]. Previous research found that the abundance of cDNA from several pathogens (bacteria, fungi, and a parasitic flatworm) was greater in pooled-transcriptome samples from the stream-spawning ecotype compared with the beach-spawning ecotype in Okanagan Lake, British Columbia (BC), Canada [8]. Moreover, a subsequent genomic scan of kokanee in four lakes across BC identified genes involved with pathogen resistance as being putatively under divergent selection between stream and beach-spawning kokanee [9]. These data suggest that kokanee may experience asymmetrical pathogen infection between spawning environments, however the lack of information on pathogen diversity within each spawning habitat has precluded a direct test of this hypothesis.

In this study, we measured the diversity and abundance of the gram-negative bacteria, *Flavobacterium sp.,* infecting Okanagan Lake kokanee from their two divergent spawning habitats (streams and beaches). *Flavobacterium* was chosen for this study because its abundance was highly correlated with ecotype in pooled transcriptome samples [8], and because several *Flavobacterium* species are associated with high levels of salmonid mortality [10], with devastating economic impacts [11–13].

## Methods

This study focused on Okanagan Lake, which is a long (135-km) and narrow (<5 km) post-glacial lake located in the southern interior of British Columbia, Canada. The large size of the lake (~350 km^2^) supports several spawning populations of both reproductive ecotypes [14, 15]. Current estimates suggest that there are ~200,000 spawning adult kokanee in Okanagan Lake. Kokanee are semelparous and philopatric, with the majority of adults spawning at an age of three years [15]. Beach spawning has been observed at most undeveloped areas of the shoreline. Stream-spawning is currently monitored at 18 tributaries, of which 60% of stream spawning occurs at a single location (Mission Creek) [15]. Stream and beach-spawning habitats experience differences in many abiotic factors such as seasonal temperature, turbidity, and rate of water-flow, which may affect the diversity of pathogens present in the two environments.

### *Flavobacterium* diversity

In order to infer the diversity of *Flavobacterium* species infecting kokanee salmon, tissue samples from spawning adult kokanee were collected from four stream and three beach-spawning sites in September 2010 (Table 1). These seven sites were chosen because they are annually monitored for kokanee abundance by the British Columbia Ministry of Forest, Lands and Natural Resource Operations. All tissue samples were collected in partnership with the British Columbia Ministry of Forests, Lands and Natural Resource Operations (collection permit PE10-66394), and in accordance with animal care protocol A11-0127 as approved by the University of British Columbia’s Animal Care & Biosafety Committee, which governs the ethical collection of specimens for research. Detailed collection information for these samples has been previously reported by Lemay *et al.* [8]. Genomic DNA was extracted from the subcutaneous muscle tissue of one individual kokanee from each of these seven sites using a Macherey-Nagel single column extraction kit following the manufacturer’s recommended protocol.

**Table 1:** **Samples used for each assay**

Spawning habitat	Sample location	1. *Flavobacterium*abundance:	2. *Flavobacterium*diversity:
		Number of kokanee sampled	Number of colonies sequenced (number of haplotypes observed)
Stream	Mission Creek	12	14 (7)
	Penticton Creek	12	12 (2)
	Peachland Creek	12	12 (2)
	Powers Creek	12	12 (4)
	**Total**	**48**	**50**
Beach	Northeast	8	15 (2)
	Northwest	20	15 (3)
	Southeast	20	20 (4)
	**Total**	**48**	**50**

(1) The number of kokanee from each location used for the qPCR assay testing the relative proportion of *Flavobacterium sp.* DNA infesting the host tissue; (2) The number of cloned *Flavobacterium sp.* amplicons that were sequenced from each sample location, and the number of different haplotypes observed at each location (in parentheses). Reads were cloned from genomic DNA extracted from a single kokanee at each sample location.

*Flavobacterium* DNA was isolated from each kokanee DNA sample using primers designed to amplify a ~200 nucleotide region of the gene encoding the ATP synthase alpha subunit (*AtpA*) of all species within the genus *Flavobacterium* [*Fspp1_F*: 5′-TTRTTAAGAAGACCACCRGG-3′, *Fspp1_R*: 5′- GGRATATATGCAGAAACGTCACC-3′]. This region was chosen because *AtpA* is part of a panel of genes used for strain identification in the species *Flavobacterium psychrophilum* [16], allowing comparisons with previously published sequence data.

Each PCR contained 2 μl DNA, 2.5 μl 10X PCR buffer, 2.5 μl dNTPs (2 mM), 1.0 μl forward primer (10 μM), 1.0 μl reverse primer (10 μM), and Ampli*Taq* Gold polymerase (0.5 units, Applied Biosystems) in a 25 μl total volume. Touchdown PCR protocol [17] was used with initial denaturation of 94°C for 10 minutes, then 10 cycles at 94°C for 30 seconds, 60°C for 30 seconds, 72°C for 60 seconds, with the annealing step decreasing by 1°C per cycle to 50°C. The annealing temperature was maintained at 50°C for an additional 30 cycles, followed by extension at 72°C for 2 minutes. All PCR products were purified using a Qiagen MinElute kit, diluted to 4 ng/μl, and ligated overnight at 4°C using the pGEM®-T Easy Vector System (Promega). Transformed cells were added to plates containing 100 μg/ml ampicillin, 0.5 mM IPTG, and 80 μg/ml X-Gal, and incubated for 16-20 hours at 37°C. White colonies were then boiled at 100°C for 10 minutes in 100 μl of TE buffer. We amplified cloned inserts from 100 colonies (50 from each ecotype) using *T7* and *Sp6* primers (Promega). Each PCR contained 1 μl colony boil, 1.25 μl 10X PCR buffer, 1.25 μl dNTPs (2 mM), 0.5 μl each primer (10 μM), and KAPA*Taq* polymerase (0.5 units; KAPA Biosystems) in a 13.5 μl total volume. Each PCR had an initial denaturation of 94°C for 2 minutes, followed by 35 cycles at 94°C for 30 seconds, 50°C for 30 seconds, 72°C for 30 seconds, with a final extension at 72°C for 2 minutes. Sequencing was carried out in one direction using *Sp6* on an Applied Biosystems 3130XL. Raw sequences were edited using Sequencher v. 5; five sequences were discarded from further analyses due to low quality and ambiguous bases.

A phylogenetic approach was then used to quantify the diversity of *AtpA* haplotypes among the remaining 95 sequences (47 stream, 48 beach; Additional file 1). We retained all distinct haplotypes irrespective of the number of reads matching the sequence. Unique haplotypes from each spawning habitat (streams and beaches) were unambiguously aligned with the *AtpA* region from the published genomes of nine *Flavobacterium* species (*F. aquatile* [GenBank: JX25684.1], *F. branchiophilum* [GenBank: NC016001.1], *F. chungnamense* [GenBank: JX256869.1], *F. columnare* [GenBank: CP003222.2]*, F. frigidarium* [GenBank: HM443893.1], *F. indicum* [GenBank: NC017025.1], *F. johnsoniae* [GenBank; NC009441.1], *F. koreense* [GenBank: JX356867.1], *F. psychrophilum* [GenBank: NC009613.1]) using MUSCLE as implemented in Geneious v.6.1 (Biomatters). This alignment was then used to generate an unrooted neighbor-joining tree in Geneious v.6.1 (Biomatters); 1000 Bootstrap replicates were performed with a 50% support threshold.

### *Flavobacterium*abundance

Quantitative PCR (qPCR) was used to measure the relative abundance of *Flavobacterium spp.* present in kokanee sampled from Okanagan Lake. This assay was carried out using DNA extracted from operculum tissue of spawning adult kokanee collected in 2007 (n = 48) and 2010 (n = 48). All kokanee samples had been collected as part of previous research [7], and included samples from both ecotypes at seven different locations in Okanagan Lake (Table 1).

The total quantity of extracted DNA (fish and pathogen) was first determined for each sample using the Quant-iT™ Pico Green ds DNA Assay Kit (Invitrogen) run on a ViiA7 real-time PCR machine (Life Technologies). Quantitative PCR was carried out using the same *AtpA* primers described above to quantify the pathogen component of each DNA sample using an absolute quantification protocol on a ViiA7 real-time PCR machine (Life Technologies). Using the same primers as the assay for *Flavobacterium* diversity allows us to quantify the abundance of all documented haplotypes. To construct the standard curve we used *F. psychrophilum* DNA of known strain and concentration [Strain: CIP103534(T), isolated from coho salmon, *Oncorhynchus kisutch*]. For increased precision, three replicates of each concentration in the standard curve were used. Each PCR contained 1 μl of DNA template, 0.5 μl of 1 μM *Fspp1_F* forward primer, 0.5 μl of 10 μM *Fspp2_R* reverse primer, and 5.0 μl of Fast SYBR® Green Master Mix (Applied Biosystems) in a total volume of 10 μl. A two-step cycling protocol was carried out with an initial denaturation of 94°C for 2 minutes, followed by 55 cycles at 94°C for 30 seconds and 60°C for 30 seconds. A melt curve stage was added to the end of the protocol beginning at 60°C and increasing to a final temperature of 98°C.

For each individual kokanee, the inferred quantity of *Flavobacterium spp.* amplicons was normalized to the total DNA template concentration of the sample in order to derive a measure of pathogen infection per unit of kokanee DNA [18]. Deviations from normality in this response variable (ng of *Flavobacterium* DNA per ng of kokanee DNA) precluded the use of parametric statistics; instead we tested for differences in *Flavobacterium* abundance between ecotypes from each sampling year using non-parametric Kruskal-Wallis tests implemented in R version 3.0.1 [19]. In addition, results of the qPCR assay were visualized using box plots generated in R using the default parameters for generating whisker lengths and designating outliers.

## Results and discussion

We tested for differences in the abundance and diversity of the salmonid pathogen, *Flavobacterium spp.*, infecting kokanee salmon from two different spawning habitats (streams and beaches). Both of the molecular assays carried out in this study (DNA sequencing and qPCR) found evidence for the presence of *Flavobacterium* from all study-sites and both spawning environments in Okanagan Lake.

**Figure 1:**
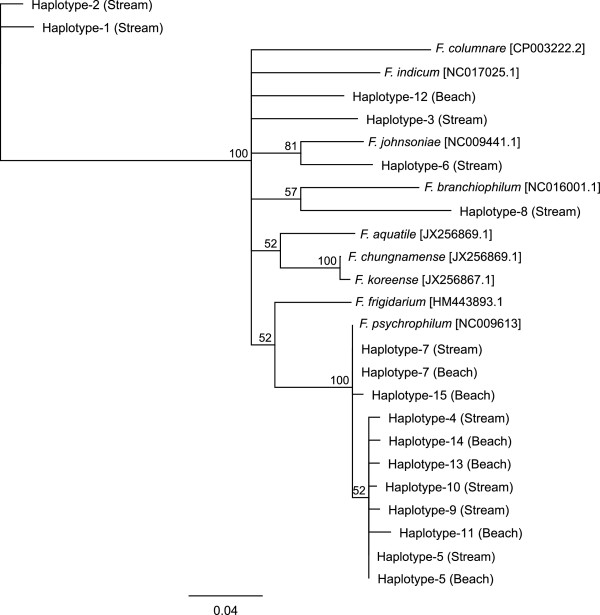
***Flavobacterium*** **diversity.** Unrooted neighbor-joining tree showing the relationship between the unique *Flavobacterium AtpA* haplotypes amplified from each ecotype (n = 10 stream, n = 7 beach). Nine previously published reference sequences are included in the phylogram with Genbank accession numbers included next to species names. Branch labels are bootstrap support values (%). Pair wise nucleotide distances between haplotypes are provided in Additional file 2.

**Figure 2:**
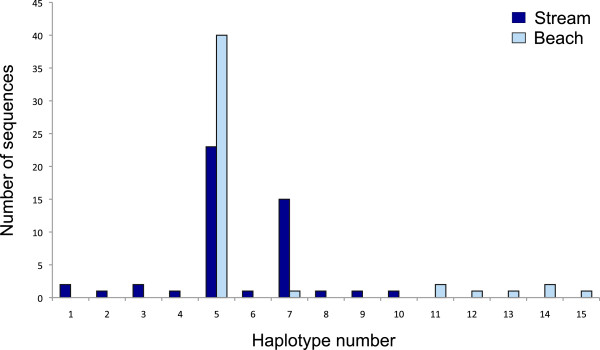
**Haplotype frequency distribution.** A frequency distribution showing the number of reads from each spawning habitat (n = 47 stream and n = 48 beach) that corresponds with each of the 15 haplotypes observed in this study. Haplotypes 4, 5, 7, 9, 10, 11, 13, 14, 15 occur within the same clade as the *F. psychrophilum* reference sequence.

Using a phylogenetic approach, we identified 10 *Flavobacterium AtpA* haplotypes among the stream-spawning kokanee and seven haplotypes among beach-spawning kokanee, with only two haplotypes shared between spawning habitats (Figures 1 & 2, Additional file 1). Haplotypes occurring in the same clade as *F. psychrophilum* were the most prevalent, occurring in kokanee from both spawning habitats (streams and beaches) and accounting for 60% of all haplotypes and 92% of all sequenced reads. The two most abundant haplotypes (haplotype 5 & 7) only differ from each other by a single nucleotide difference and account for 82% of all sequenced reads; with Haplotype-7 identical to the reference sequence for *F. psychrophilum* (Additional file 2)*.* Haplotypes that fall on clades other than that of *F. psychrophilum* are only represented by 1-2 reads per haplotype, and none of these remaining haplotypes were shared between spawning habitats.

**Figure 3:**
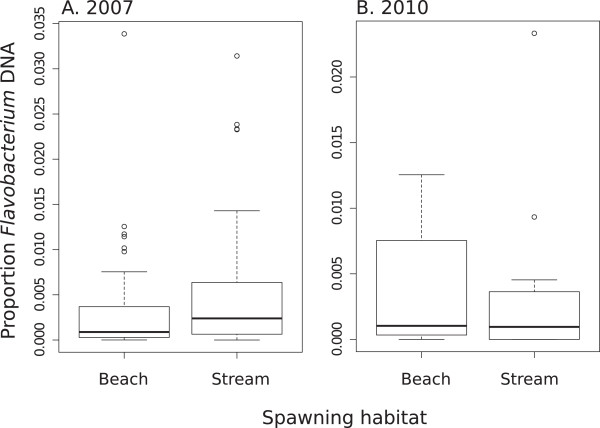
***Flavobacterium*** **abundance.** Relative abundance of *Flavobacterium AtpA* haplotypes amplified from the operculum tissue of kokanee sampled at stream and beach spawning sites in Okanagan Lake based on qPCR assays. Samples are separated by year: **(A)** 2007 (n = 48); **(B)** 2010 (n = 48).

The results from the qPCR assay did not reveal any significant difference in the relative abundance of *Flavobacterium AtpA* amplicons between samples from stream- and beach-spawning sites (2010 *p* = 0.211; 2007 *p* = 0.204; Figure 3).

The phylogenetic results provide preliminary evidence that there may be differences in the composition of Flavobacteria species/strains between the two spawning habitats, however the small sample size (3-4 kokanee per spawning habitat) severely limits our ability to draw definitive conclusions. For example the small sample sizes may skew the observed distribution of rare haplotypes. Yet, the relatively high diversity of *Flavobacterium AtpA* haplotypes observed in the tissue of only seven kokanee samples provides evidence that individuals may be infected by multiple strains and/or species of *Flavobacterium*, however the small size of the amplicon (~200 base pairs) precludes determination of species identity.

It would be highly informative for future research to sequence larger regions and additional genes in order to determine the identity of species present. Alternatively, the use of high-throughput methods for quantifying microbial community assemblages, such as 16 s rRNA gene sequencing [20], could be used to provide insight into the composition of bacterial communities inhabiting kokanee from each spawning habitat. This approach has been used to quantify microbial communities in a diversity of study systems including plants [21], animals [22, 23], and soils [24], and provides an effective method to test for differences in bacterial community structure. For example, in Atlantic salmon, *Salmo salar*, 16 s rRNA gene analysis was used to identify bacterial communities putatively associated with infectious amoebic gill disease [25].

In order to better understand the ecological interactions between kokanee and their bacterial pathogens, it would also be useful for future research to examine *Flavobacterium* diversity and abundance from environmental samples in the two habitats. While *F. psychrophilum* is both horizontally and vertically transmitted to new hosts [26, 27], it can also persist for long periods of time (300 days) without a host [28]. Therefore, the compliment of pathogens infecting fish tissues may not be an accurate estimate of the total diversity and abundance present in each habitat.

## Conclusions

Genes associated with immune response are often identified as candidate regions for local adaptation in salmonids [29–33], including in kokanee [9], and it has been shown that patterns of divergence at these genes may result from localized differences in pathogen diversity across fine spatial or temporal scales [34–37]. While this current study only found weak evidence for differences in pathogen diversity in kokanee collected from different spawning habitats (streams and beaches), these results warrant further study. Future research aimed at quantifying pathogen diversity and abundance in population-level samples of kokanee combined with environmental sampling is needed to better understand the ecology of pathogen infection in this system.

### Availability of supporting data

The data sets supporting the results of this article are included within the additional files.

## Electronic supplementary material

Additional file 1: **Flavobacterium AtpA haplotype sequences.** A list of the unique *AtpA* haplotypes used to generate Figure 1. Sections of the NCBI reference sequences from previously published Flavobacteria species are also included. (ZIP 989 bytes)

Additional file 2: **Pair wise distance matrix.** A pair wise matrix showing the number of nucleotide differences between each *AtpA* haplotype described in Figures 1 & 2. (CSV 3 KB)
